# Ginsenoside Rh2 Suppresses Metastasis and Growth of Colon Cancer via miR-491

**DOI:** 10.1155/2021/6815713

**Published:** 2021-09-24

**Authors:** Wene Wei, Qijing Guo, Cuiping Guo, Xianshu Cui, Xuemei Ma, Xianliang Shen, Yushuang Luo

**Affiliations:** Department of Oncology, Qinghai University Affiliated Hospital, Xining 810001, Qinghai, China

## Abstract

Ginsenoside Rh2 is considered as a new direction for future cancer treatment because of its excellent anticancer effect. However, due to its low bioavailability, it cannot exert its significant anticancer effect when applied directly to the human body. Chitosan (CS), a nanomaterial, has been verified to be able to enhance drug efficacy via its coating for drugs. Thus, we designed this study to investigate the impact of CS-coated ginsenoside Rh2 on the metastasis and growth of colon cancer (CC). First, ginsenoside Rh2 chitosan tripolyphosphate (CS-Rh2-TPP) nanoparticles (NPs) were constructed, and MTT, transwell, scratch adhesion, and flow cytometry assays were carried out for determining the impact of CS-Rh2-TPP at various concentrations on growth, metastasis, and apoptosis of colon cancer cells (CCCs). qRT-PCR was used to detect the expression of mircoRNA-491 (miR-491) in CCCs. According to TEM-based image analysis, CS-Rh2-TPP NPs were spherical or spheroidal in even distribution, with a particle size of about 220 mm and a zeta potential of −44.58 ± 2.84 mV. Additionally, CCCs presented lower miR-491 than normal colon cells, and its relative expression in CCCs showed a stronger increase after intervention of CS-Rh2-TPP than that after intervention of ginsenoside Rh2. Moreover, CS-Rh2-TPP suppressed the activity, invasion, as well as migration of CCCs and accelerated their apoptosis more significantly than ginsenoside Rh2. According to these results, CS-Rh2-TPP is able to upregulate miR-491 in CCCs, thus suppressing the metastasis and growth of CC.

## 1. Introduction

Due to changes in diet and living structure, gastroenterological diseases present a rising incidence, and colon cancer (CC) is a common one with a comparatively high global incidence [1]. The survey shows that the global incidence of CC is about 6.1% at present. Each year witnesses over 1 million new cases of CC and over 550,000 new deaths from the cancer [2]. Therefore, clinical efforts have been devoted to find solutions. The unfavorable prognosis of patients with CC is mainly caused by the difficulty in early clinical screening of CC and the lack of special clinical symptoms of early CC [3]. In fact, most patients have already entered the middle or late stage at the time diagnosis due to their lack of medical and health knowledge, resulting in the missing of the optimal timing for surgical treatment [4]. At this time, the tumor is usually accompanied by metastasis and invasion, and the commonly used clinical treatment schemes (surgery or combined chemoradiotherapy) generally cannot achieve the best effect of tumor resection [5].

Ginsenoside Rh2 is a primary active substance extracted from Ginseng, with potent pharmacological effects [6]. Its clinical impacts known so far include immunomodulatory activity and increasing cognitive ability [7]. It also plays a crucial part in antioxidant and antitumor activities [8]. According to studies, ginsenoside Rh2 suppresses angiogenesis in patients with prostate cancer through targeting CNNM1 [9] and accelerates the apoptosis of cervical cancer cells during starvation [10]. Additionally, ginsenoside Rh2 has been verified to affect tumors via miRs [11]. MicroRNA (miR) is a research focus in various fields. As a noncoding short-chain RNA in eukaryotes with about 22 nt in length, miR can regulate many intercellular signals via regulation on target genes by binding to downstream target genes 3' UTR, 5' UTR, and coding regions [12]. miR-491, a newly discovered miR, shows low expression in cases with CC according to one early study [13]. Another study [14] revealed that ginsenoside Rh2 can inhibit the growth of lung cancer (LC) via miR-491.

Thanks to the continuous advancement of medical technology, nanomedicine has attracted extensive application in clinical practice [15]. As a polymer colloid particle system (10–500 nm in diameter), NPs have been extensive used in carrying and delivering bioactive substance [16]. Chitosan (CS) is a biomaterial made by deacetylation of chitin from shrimps and crab, with favorable antibacterial and antiviral properties [17]. In one recent study, ginsenoside Rh2-CS NPs have been revealed to suppress the activity of LC cells [18]. However, whether it possesses the same effect in CC is still under investigation.

Accordingly, this study primarily investigated the impact of ginsenoside Rh2-CS NPs on the growth and metastasis of colon cancer cells (CCCs).

## 2. Materials and Methods

### 2.1. Preparation of CS NPs

The preparation of ginsenoside Rh2 chitosan tripolyphosphate (CS-RH2-TPP) nanoparticles (NPs) was carried out by referring to the study of Zare-Zardini et al. [19]. CS (4 mg, Shandong AK Biotech Co., Ltd., China) was stirred in 1% acetic acid (4 mL, Sigma-Aldrich, Merck KGaA) until it was completely dissolved, and then, the PH was adjusted to 5 with 2 mol/L NaOH. Ginsenoside Rh2 (1.2 mg, Zhejiang Yake Pharmaceutical Co., Ltd., China) was weighted and dissolved in methanol, which was then dripped into CS solution at 20 drops/min. Afterwards, equal volume of TPP solution was added dropwise and stirred for 30 min crosslinking reaction, followed by filtering via a 0.45 *μ*m filter membrane and freeze drying. Finally, the obtained substance was stored at 4°C.

### 2.2. Identification of CS NPs

A transmission electron microscope (TEM, Beijing Precise Instrument Co., Ltd., China) was adopted to observe the microscopic morphology of CS NPs. Specifically, 1.0 mL solution was put on a carbon-coated copper grid, restained with phosphotungstic acid (Sigma-Aldrich, Merck KGaA), and then evaluated under a TEM after drying. Subsequently, 1.5 mL solution was made to penetrate a 0.22 *μ*m microporous membrane, followed by analysis via a Malvern particle size analyzer (Zhuhai OMEC Instruments Co., Ltd., China) for understating the particle size and distribution.

### 2.3. Cell Culture

SW480 and SW620 cells (CCCs) were selected, and FHC cells (normal human colonic epithelial mucosal cells) were adopted as controls, all of which were offered by the American Type Culture Collection. The above cells were incubated (37°C, 5%CO_2_) in 10% fetal bovine serum (FBS) + 1% penicillin/streptomycin-contained DMEM (Gibco).

### 2.4. MTT Assay

Cell viability was detected by MTT assay. Specifically, the transfected cells were transferred to a 96-well plate (2^*∗*^10^5^ cells/well) after resuspension and cultured at room temperature for 24 h. Then, the plate was treated by 24 h of incubation with CS-Rh2-TPP or ginsenoside Rh2 at various concentrations (5, 10, and 20 *μ*g/mL), followed by 4 h of incubation with 20 *μ*L MTT complete medium per well (Thermo Fisher Scientific, USA), as well as 10 min mixing at 492 nm with complete medium replaced by 200 *μ*L DMSO.

### 2.5. Transwell Assay

A transwell assay (Corning, USA) was conducted to understand the impact of CS-Rh2-TPP/ginsenoside Rh2 on the invasion of CCCs. Specifically, a Matrigel-coated transfer chamber with an 8 *μ*m porous polycarbonate membrane was adopted. Totally, 100 *μ*L DMEM (serum-free) suspended with 5 × 10^4^ cells were transferred to 500 *μ*L FBS-contained DMEM together with cell culture insert, followed by 24 h of incubation with CS-Rh2-TPP or ginsenoside Rh2 at various concentrations (5, 10, and 20 *μ*g/mL). Afterwards, noninvasive cells on the upper surface of the membrane were scraped off, while the remaining was treated by immobilization via 4% paraformaldehyde and dyeing with 1% crystal violet. Finally, under an optical microscope, cells in a randomly selected area were counted.

### 2.6. Wound-Healing Assay

To investigate the impact of CS-Rh2-TPP/ginsenoside Rh2 on the migration of CCCs, a wound-healing assay was conducted. Specifically, in a 6-well plate seeded with 4 × 10^5^ cells, two parallel wounds were created in the cell monolayer with a 10 *µ*l pipette tip at the cell confluence of over 90%. Then, PBS was adopted for twice washing of the cell fragments that were then subjected to incubation in serum-free DMEM and then to 24 h of incubation with CS-Rh2-TPP or ginsenoside Rh2 (5, 10, and 20 *μ*g/ml) at different concentrations. Finally, cell images were taken with one optical microscope (×200), followed by calculation of the wound width by a standard caliper.

### 2.7. Flow Cytometry Assay

The impact of CS-Rh2-TPP/ginsenoside Rh2 on the apoptosis of CCCs was determined with an Annexin V-APC kit (Shanghai Yeasen Biotechnology Co., Ltd., China). CS-Rh2-TPP or ginsenoside Rh2 at various concentrations (5, 10, and 20 *μ*g/mL) were put into CCCs for 24 h of incubation, and the collected cells were determined via a FACS Calibur flow cytometer (BD Biosciences). CCCs collected via EDTA-free trypsin were suspended in binding buffer after two times of washing with cold PBS, followed by 15 min of dyeing with Annexin V-FITC and propidium iodide (PI). Finally, the proportion of apoptotic cells (Annexin V-FITC positive) in the total number of counted cells was calculated.

### 2.8. qRT-PCR Assay

After extraction of total RNA from collected cells with a TRIzol kit (Thermo Fisher Scientific, USA), its concentration, purity, as well as integrity were confirmed with an ultraviolet spectrophotometer and agarose gel electrophoresis. Then, the total RNA was reverse transcripted to complementary DNA (cDNA) in strict accordance with the kit (Thermo Fisher Scientific, USA) instructions. The amplification system: 1 *μ*L cDNA, 0.4 *μ*L upstream and downstream primers, respectively, 10 *μ*L 2 × TransTaq® Tip Green qPCR SuperMix, 0.4 *μ*L passive reference dye (50X), and ddH_2_O added for volume adjustment (20 *μ*L in total); the amplification conditions: conditions for PCR reaction: 94°C for 30 s, followed by 40 cycles of 94°C for 5 s and 60°C for 30 s. Each sample was determined three times with three duplicate wells, and the obtained data were analyzed via 2^−△△ct^ (internal reference of miR: U6).

### 2.9. Statistical Analyses

In our study, GraphPad 8 was adopted for data analysis and data visualization into corresponding figures. Measurement data were presented by mean ± SD; the intergroup comparison was carried out via the independent-samples *t*-test, and the multigroup comparison was conducted by the one-way ANOVA (expressed in F) and LSD-t post hoc test. Additionally, the comparison of data at various time points was performed by the repeated measures ANOVA (expressed in F), and their post hoc comparison by the Bonferroni post hoc test. *P* < 0.05 denotes a remarkable difference.

## 3. Results

### 3.1. Identification of CS NPs

**Figure 1:**
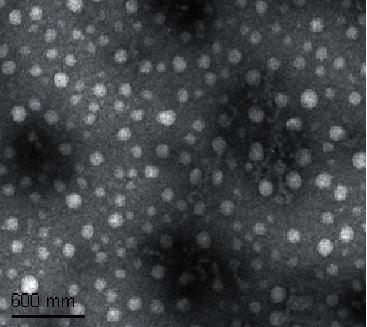
CS-Rh2-TPP nanoparticle morphology.

In our study, the constructed CS-Rh2-TPP NPs were identified first. According to TEM-based image analysis, CS-Rh2-TPP NPs were spherical or spheroidal in even distribution, with a particle size of 220 mm (Figure 1), negatively charged surface, and zeta potential of −44.58 ± 2.84 mV.

### 3.2. MiR-491 in CCCs

**Figure 2:**
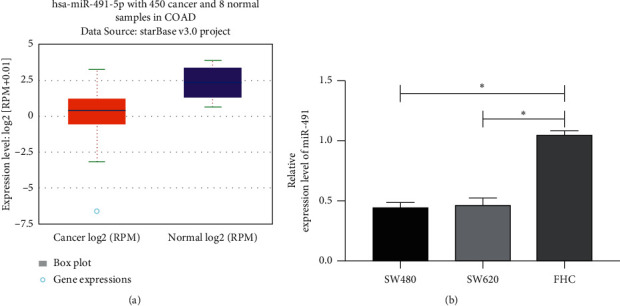
MiR-491 in CC (a). MiR-491 in cases with CC according to online analysis based on TCGA (b). MiR-491 in CCCs according to qRT-PCR assay. ^*∗*^*P* < 0.05. Note: CS-Rh2-TPP, ginsenoside Rh2 chitosan tripolyphosphate; CCCs, colon cancer cells; CC, colon cancer.

We analyzed the expression of miR-491 in CCCs based on the TCGA database through Starbase online and found that it was downregulated in CCCs (Figure 2(a)). We also quantified miR-491 in CCCs via a qRT-PCR assay. It was found that the expression of miR-491 in CCCs was significantly lower than that in normal colon cells, which indicates its low expression in cases with CC (Figure 2(b)).

### 3.3. Impact of CS-Rh2-TPP on miR-491 in CCCs

**Figure 3:**
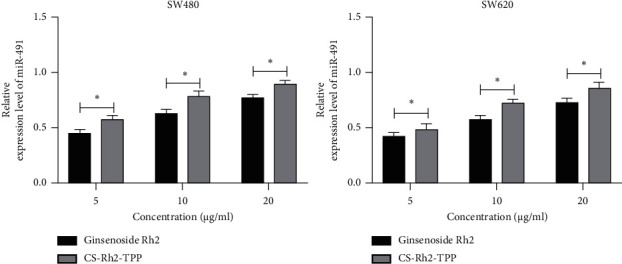
Impact of CS-Rh2-TPP on miR-491 in CCCs. ^*∗*^*P* < 0.05. Note: CS-Rh2-TPP, ginsenoside Rh2 chitosan tripolyphosphate; CCCs, colon cancer cells.

We have quantified miR-491 in cases with CC through the above assay. Then, we evaluated the impact of CS-Rh2-TPP/ginsenoside Rh2 on miR-491 in CCCs. According to the qRT-PCR assay, miR-491 in cases with CC increased more notably with the increase of their concentration, and the increase was more significant under the intervention of CS-Rh2-TPP than that under the intervention of ginsenoside Rh2 (Figure 3). The results suggest that CS-Rh2-TPP has a more significant effect on miR-491 in CCCs.

### 3.4. Impact of CS-Rh2-TPP on Cell Activity

**Figure 4:**
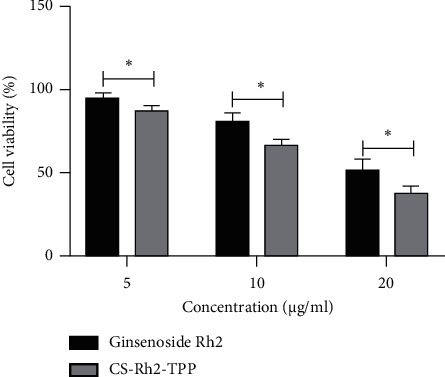
Suppression of CS-Rh2-TPP on viability of CCCs. ^*∗*^*P* < 0.05. Note: CS-Rh2-TPP, ginsenoside Rh2 chitosan tripolyphosphate; CCCs, colon cancer cells.

For the purpose of exploring the impact of CS-Rh2-TPP on cell activity, we adopted CS-Rh2-TPP/ginsenoside Rh2 at various concentrations to intervene with CCCs. According to the MTT assay, the cell viability was more notably suppressed as their concentrations increased, and the suppression was stronger under intervention of CS-Rh2-TPP than that of ginsenoside Rh2 (Figure 4).

### 3.5. Impact of CS-Rh2-TPP on Cell Invasion and Migration

**Figure 5:**
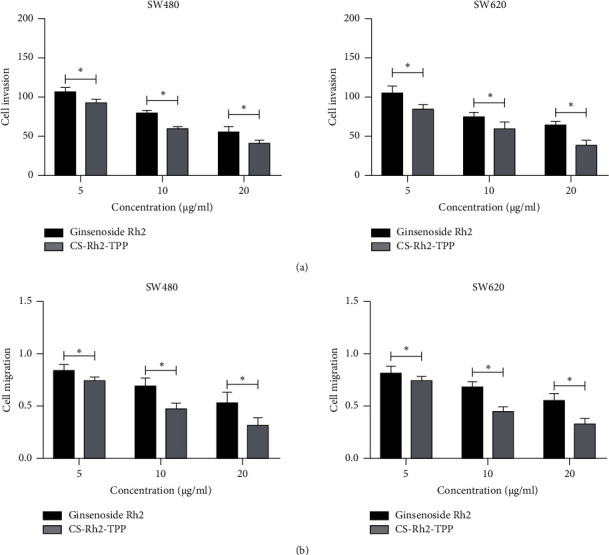
Impact of CS-Rh2-TPP on invasion and migration activities of CCCs (a). Impact of CS-Rh2-TPP/ginsenoside Rh2 on invasion of CCCs according to the transwell assay (b). Impact of CS-Rh2-TPP/ginsenoside Rh2 on migration of CCCs according to the wound-healing assay. ^*∗*^*P* < 0.05. Note: CS-Rh2-TPP, ginsenoside Rh2 chitosan tripolyphosphate; CCCs, colon cancer cells.

This study also evaluated the impact of CS-Rh2-TPP/ginsenoside Rh2 on the invasion and migration activities of CCCs. According to assay results, under intervention of CS-Rh2-TPP/ginsenoside Rh2, the invasion and migration activities of CCCs were increasingly inhibited as the concentration of CS-Rh2-TPP/ginsenoside Rh2 increased. Moreover, the intergroup comparison showed that the effect of CS-Rh2-TPP on the invasion and migration activities of CCCs was stronger than that of ginsenoside Rh2 (Figures 5(a) and 5(b)).

### 3.6. Impact of CS-Rh2-TPP on Apoptosis of CCCs

**Figure 6:**
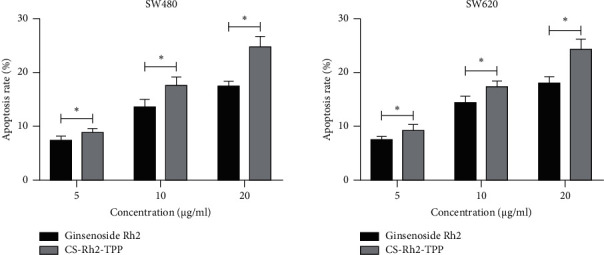
Impact of CS-Rh2-TPP on apoptosis of CCCs. ^*∗*^*P* < 0.05. Note: CS-Rh2-TPP, ginsenoside Rh2 chitosan tripolyphosphate; CCCs, colon cancer cells.

We also evaluated the impact of CS-Rh2-TPP on the apoptosis of CCCs. According to assays, the apoptosis of CCCs increased more notably as the concentration of CS-Rh2-TPP/ginsenoside Rh2 increased, and CS-Rh2-TPP exerted a more notable promotion effect on the apoptosis of CCCs than ginsenoside Rh2 (Figure 6).

## 4. Discussion

CC is the most common digestive tract malignancy, but its mechanism is still under investigation. In our study, ginsenoside Rh2 suppressed the growth and metastasis of CCCs and the constructed CS-Rh2-TPP exerted a more notable inhibitory action, so CS-Rh2-TPP is expected to be a clinical therapy scheme for CC.

Ginseng is a Chinese herbal medicine widely found in Asian countries, with various beneficial properties, including anti-inflammatory, antioxidation, and anticancer activities [20]. Ginsenoside Rh2, the primary active extract of Ginseng, is considered as a broad anticancer agent [21]. According to one study [22], ginsenoside Rh2 has the advantages of low toxicity, low molecular weight, and good fat solubility. And its ability to inhibit the proliferation and migration of tumor cells and angiogenesis has been well documented [23]. One study [24] reported that ginsenoside Rh2 affected tumorigenesis through regulating encoded proteins or encoded RNAs [24]. MiR-491, a newly discovered miR, has been shown to inhibit the metastasis and growth of LC [25], bladder cancer [26], and gastric cancer [27]. One study by Lu et al. [28] revealed the anticancer role of miR-491 in colorectal cancer by targeting IGF2 [28]. In our study, ginsenoside Rh2 did lower the activity of CCCs, and under its intervention, the cells presented higher miR-491 and weaker growth and metastasis activities. The results suggest the ability of ginsenoside Rh2 to regulate the metastasis and growth of CCCs via miR-491.

Nanomedicine is one of the research hotspots in recent years and has made outstanding contributions to medical therapy and diagnosis [29]. Drugs coated by nanomaterials have been found to have higher efficacy and less loss during circulation [30]. For instance, solid lipid NPs and those coated by CS are promising tools for silybin delivery [31], and methotrexate-loaded fucoidan/CS NP has anti-inflammatory potential and enhanced skin permeability [32]. CS, as the product of N-deacetylation of chitin, has the advantages of nontoxicity, bacteriostasis, lipid-lowering, and anticancer properties. Reportedly, drugs coated by CS possess stronger efficacy against cancer than such drugs without coating [33]. For instance, doxorubicin/cisplatin combined with hyaluronic acid/CS NPs boosts the efficacy of synergistic combination chemotherapy for breast cancer [34]. CS-capped ZnO NPs have specific apoptosis induction ability via P53 activation and G2/M arrest in breast cancer cells [35]. In our study, we constructed CS-Rh2-TPP materials and found through assays that compared with ginsenoside Rh2, CS-Rh2-TPP exerted a stronger effect in inducing the apoptosis of CCCs, suppressing their activity, migration, and invasion, and upregulating miR-491 in them. The above data show that the anticancer effect of CS-Rh2-TPP is stronger than that of ginsenoside Rh2 alone. As the disease with the highest mortality at present, the main clinical treatment of malignant tumor is still surgery or combined chemoradiotherapy, but the prognosis of patients is not optimistic. With the deepening of research, there is consensus at home and abroad that molecular antitumor will be the key to conquering tumors in the future. On this basis, more and more studies have pointed out that a variety of drugs can inhibit the occurrence and development of tumors through molecular pathways. Among them, ginsenoside Rh2 has this effect. However, ginsenoside Rh2 cannot play its excellent anticancer effect in human body due to its low bioavailability and fast metabolism. Therefore, how to solve this problem has become a hotspot and difficult point in clinical research. In this experiment, CS-RH2-TPP NPs were prepared and achieved a significantly better inhibitory effect on CC than ordinary ginsenoside Rh2. This indicates that CS-RH2-TPP NPs have great clinical application prospects in the future, which can effectively address the application limitations at the present stage and provide new ideas and directions for the treatment of CC, rendering an important guarantee for the prognosis of patients.

However, our study still has its limitations. For instance, it has just determined the impact of CS-Rh2-TPP on biological function of CCCs. In addition, it is an in vitro experiment, so whether CS-Rh2-TPP has such effect on animal models needs further experimental support. Therefore, we hope to further carry out assays to improve our conclusions in the future.

## 5. Conclusion

To sum up, CS-coated ginsenoside Rh2 can upregulate miR-491 in CCCs, thus suppressing the growth and metastasis of CC.

## Data Availability

The data used to support the findings of this study are available from the corresponding author upon request.
